# Case Report: Intraarticular Iliopsoas Tendon causes Groin Pain Following Periacetabular Osteotomy

**DOI:** 10.3389/fsurg.2022.870993

**Published:** 2022-04-27

**Authors:** Sebastian Gebhardt, Lars Nonnenmacher, Georgi I. Wassilew, Alexander Zimmerer

**Affiliations:** Center for Orthopaedics, Trauma Surgery and Rehabilitation Medicine, University Medicine Greifswald, Greifswald, Germany

**Keywords:** PAO, femoroplasty, iliopsoas tendon, impingement, pain

## Abstract

A 43-year-old female patient reported persistent iliopsoas-related groin pain following periacetabular osteotomy (PAO) combined with femoroplasty *via* a direct anterior approach due to CAM morphology. Concomitantly with the planned removal of screws, hip arthroscopy was performed, and the iliopsoas tendon was found to run intraarticularly, resulting in the tendon being impaired in its mobility and being entrapped. The tendon was arthroscopically released. The patient reported relief of the groin pain after the arthroscopic tendon debridement. During PAO combined with capsulotomy, the postoperatively observed intraarticular position of the iliopsoas tendon should be prevented by careful closure of the joint capsule.

## Introduction

Periacetabular osteotomy (PAO), first described by Ganz et al. in 1988 (1), is a well-established technique for treating hip pain caused by inadequate acetabular coverage of the femoral head. To address an existing CAM morphology that may lead to femoroacetabular impingement syndrome (FAIS) in a supplementary manner, arthroscopy of the hip joint or an open approach *via* the direct anterior approach (DAA) can be performed.

Good results are reported for PAO, with significantly increased patient-related outcome measurements (PROMs) (2), and survivor rates are 90, 60, and 30% after 10, 20, and 30 years, respectively (3, 4). However, up to 10% of patients are reported to have postoperative iliopsoas-related pain besides satisfying bony correction of the acetabulum (5). To our knowledge, this is the first report of an intraarticular position of an iliopsoas tendon after PAO with additional femoroplasty using a DAA involving a capsulotomy. Thus, this report offers feasible rationale and treatment option regarding the observed complication associated with groin pain after successful bone correction by PAO.

## Case Report

### Patient Information

A 43-year-old female patient presented to our clinic with persistent left groin pain 1 year after PAO and femoroplasty *via* the DAA for acetabular retroversion and combined FAIS. Despite taking daily nonsteroidal anti-inflammatory drugs (NSAIDs) and physical therapy, she was unable to work and was restricted in her leisure activities. In particular, she complained of pain with active hip flexion. Other than the significant groin pain, her postoperative course had been unremarkable with adequate wound healing and postoperative recovery.

### Clinical Findings

On clinical examination after the PAO, the wound was unremarkably healed, and neuromuscular function was found to be intact. The patient showed a positive flexion-adduction-internal-rotation (FADIR) test and iliopsoas tendon-related pain especially with active flexion of the hip against resistance. The internal rotation of the hip was increased to 30° compared to the 20° before PAO. Further range of motion was unchanged with external rotation 50°, extension/flexion 10-0-100°, and abduction/adduction 60-0-30°.

### Timeline

The patient reported having hip pain for years. Initial conservative measures failed; thus, given typical radiological findings, she was scheduled for PAO and concomitant femoroplasty. According to our standard of care, femoroplasty was performed through the DAA without closure of the capsule after trimming of the femoral neck. In the postoperative course, besides unremarkable wound-healing and mobilization, she reported persistence of groin pain. Consequently, we proposed concomitant hip arthroscopy with the removal of screws 1 year after the operation.

### Diagnostic Assessment

**Figure 1:**
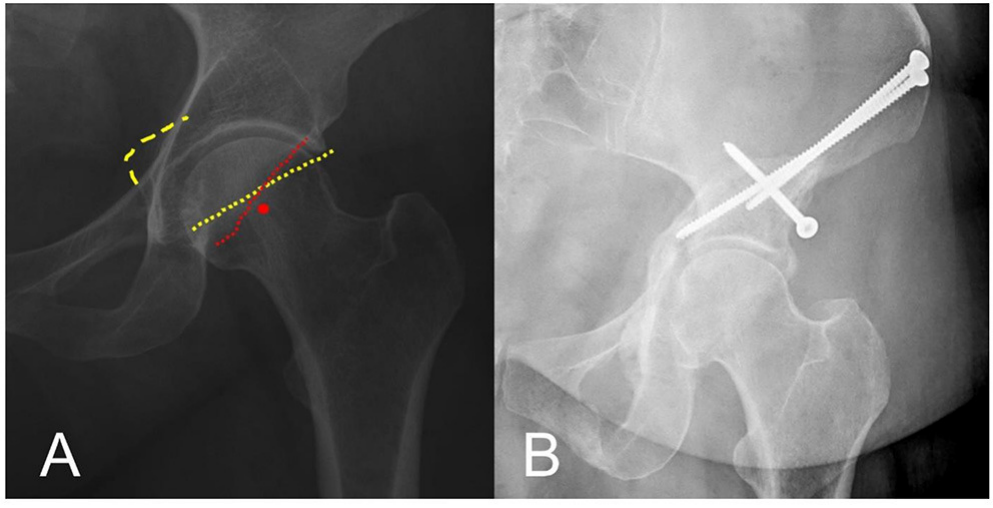
**(A)** Anterior-posterior (AP) radiograph of the left hip: signs of acetabular retroversion are present; posterior wall sign (PWS), red circle; crossover sign (COS), dotted red and yellow line; ischial spine sign, dashed yellow line. **(B)** AP radiograph of the left hip: satisfying bony correction and correct screw placement after anteverting periacetabular osteotomy (PAO) of the left hip.

**Figure 2:**
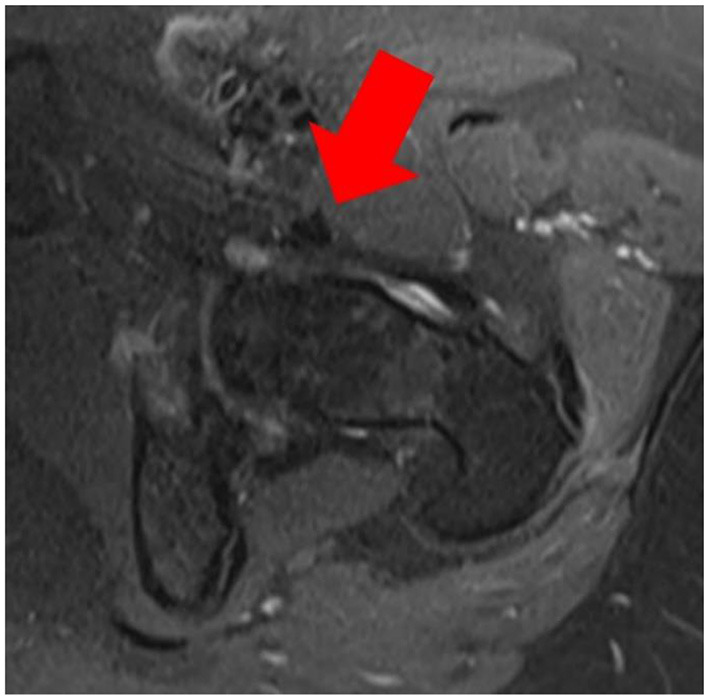
Pre-PAO MRI of the left hip shows the iliopsoas tendon running extracapsularly (red arrow).

Initial anterior-posterior (AP) pelvis and left 45°-Dunn-view radiographs before the PAO showed a crossover sign (COS), posterior wall sign (PWS), ischial spine sign, and a lateral center edge angle (LCEA) of 38° (Figure 1A). The initial magnetic resonance imaging (MRI) of the left hip showed a lesion of the labrum and an anterolateral subchondral acetabular edema, while the course of the psoas tendon was clearly extraarticular (Figure 2). The post-PAO radiograph of the pelvis obtained showed satisfying bony correction (COS and PWS not observed, LCEA 38°) and correct placement of osteosynthesis screws as well as complete consolidation of the osteotomy (Figure 1B).

**Figure 3:**
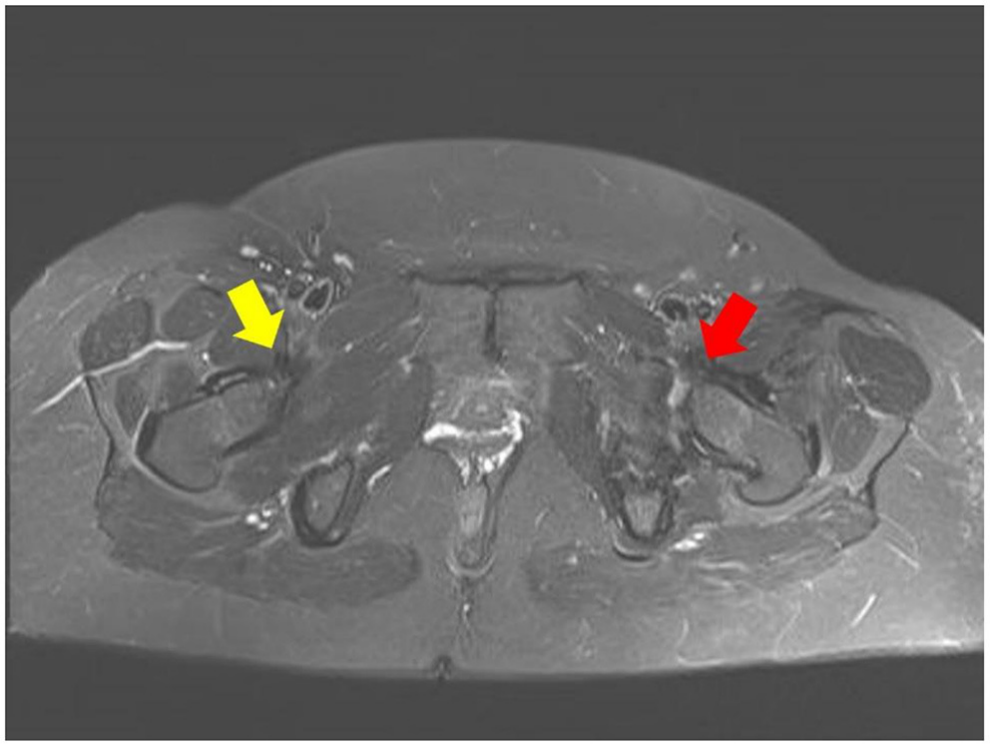
Follow-up MRI after minimal invasive PAO with anteversion of the acetabulum showing normal anatomy of the right psoas tendon (yellow arrow) and intraarticular position of the left iliopsoas tendon (red arrow).

An MRI post PAO, besides being compromised by metal artefacts, showed an intraarticular position of the psoas tendon and subsequent edema (Figure 3).

### Therapeutic Intervention

**Figure 4:**
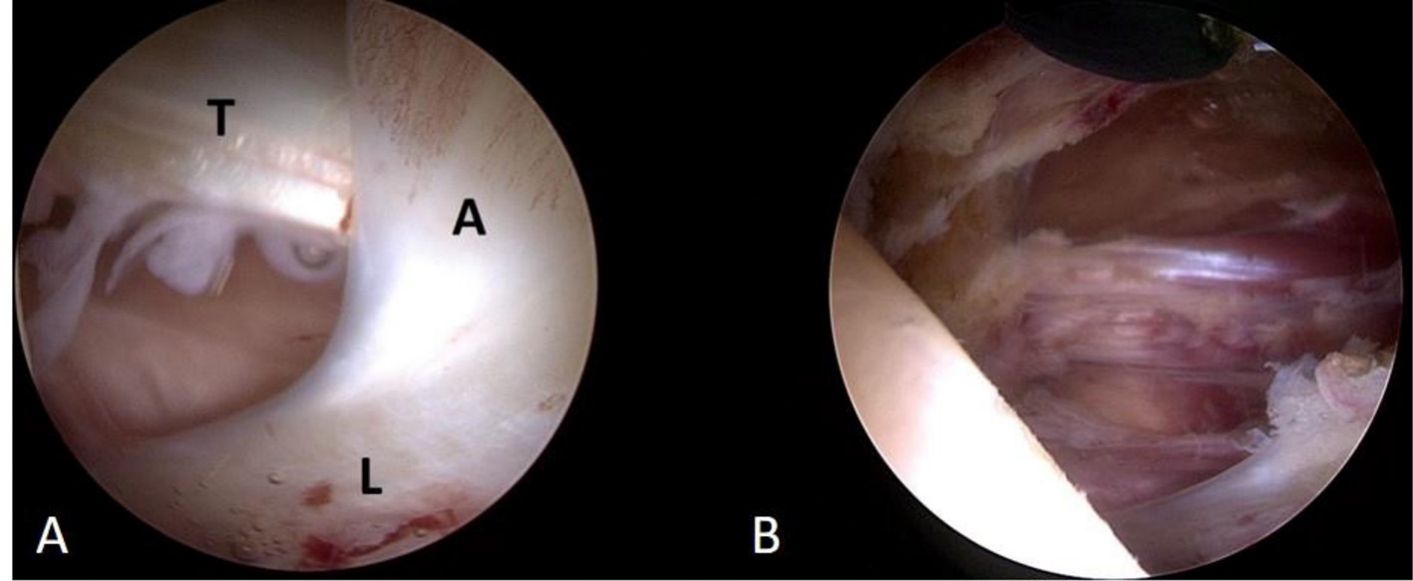
**(A)** Intraopertive view showing the labrum (L), locally inflamed adhesion (A), and intraarticular position of the iliopsoas tendon (T). **(B)** View after the arthroscopic release of the adhesion and iliopsoas tendon to prevent further impingement.

The patient was recommended to undergo hip arthroscopy in the process of operative screw removal to verify the hypothesis of iliopsoas impingement. Intraoperatively, the iliopsoas tendon was found to run intraarticularly, causing it to be trapped and limiting its mobility. The labrum showed adhesions that were locally inflamed (Figure 4). An arthroscopic release of the inflamed adhesions and debridement of the iliopsoas tendon was performed by the senior author (AZ).

### Follow-Up and Outcomes

The early postoperative course was unremarkable. The patient reported relief of the groin pain earlier described. In particular, she was able to actively flex her hip without pain within 2 weeks after iliopsoas tendon debridement.

## Discussion

The most common indication of PAO is hip dysplasia. Hip dysplasia is defined by insufficient coverage of the femoral head by the acetabulum and is considered a risk factor for the development of groin pain and early onset of arthritis of the hip. The indication for surgical treatment of hip dysplasia in adults consists of clinical and radiological findings. Patients with LCEA <2 5°, hip osteoarthritis < Tönnis grade 2, body mass index (BMI) < 30 kg/m^2^, age <45, and persistence of groin pain for > 3 month have been shown to be especially eligible for surgical treatment (6). Further indications for PAO can be pathologic acetabular retroversion resulting in FAIS. Compared with other pelvic osteotomies, PAO has been postulated to have a lower pseudarthrosis rate, which could be attributed to increased postoperative pelvic stability and larger cancellous contact surfaces (7).

The initial cohort of 75 consecutive dysplastic hips treated by Ganz showed encouraging long-term results at 11 years follow-up with 73% good to excellent results and an overall preservation rate of the joint of 82%. Major complications were only observed in the very first 18 patients (8). Recently research focused on patient-related outcome scores for younger and active patients which showed that despite significant improvement in pain level and function after PAO the average scores remained lower than for healthy age-related counterparts. A remarkably high proportion of 35% of patients experienced persistence of groin pain after PAO (6). While there is no obvious explanation for this postoperative complication, it is interesting that similar observations were made after total hip arthroplasty (THA). In patients who experienced groin pain after THA, among other factors like implant loosening, impingement of the iliopsoas tendon was identified as source of the pain (9).

The standard approach used for PAO in our clinic aims not to open the joint capsule (10); however, the capsule can either be opened accidently when preparing the osteotomy of the ilium by bluntly releasing structures off the capsule with a Hohman's retractor or intentionally for concomitant treatment of intraarticular pathologies *via* capsulotomy. The latter mentioned is similar to the DAA for THA. In this case, we hypothesize that because of the capsulotomy for femoroplasty, the iliopsoas tendon was transposed intraarticularly, and that the resulting entrapment led to the observed groin pain. As recent reports had found good results for arthroscopic iliopsoas release in cases with iliopsoas impingement after THA (11), the indication for diagnostic hip arthroscopy and iliopsoas tendon release was made in this case. The necessity of closure of the hip capsule for biomechanical reasons and superior clinical outcomes of hip joint preservation surgery have recently been discussed controversially with recommendation for closure of the hip capsule in young and active patients (12, 13). Considering the findings and the pathology presented in this case report, closure of the hip joint capsule after PAO with concomitant femoroplasty is recommended.

## Conclusion

The intra-articular position of the Iliopsoas tendon may be a cause of groin pain after PAO with concomitant open treatment of intra-articular pathology. In PAO without open hip treatment, the capsule could accidently be opened in the process of preparation of the ilium. In both cases, careful closure of the joint capsule is recommended to prevent iliopsoas impingement.

## Data Availability Statement

The original contributions presented in the study are included in the article/supplementary material, further inquiries can be directed to the corresponding author.

## Author Contributions

All authors listed have made a substantial, direct, and intellectual contribution to the work and approved it for publication.

## Conflict of Interest

The authors declare that the research was conducted in the absence of any commercial or financial relationships that could be construed as a potential conflict of interest.

## Publisher's Note

All claims expressed in this article are solely those of the authors and do not necessarily represent those of their affiliated organizations, or those of the publisher, the editors and the reviewers. Any product that may be evaluated in this article, or claim that may be made by its manufacturer, is not guaranteed or endorsed by the publisher.
